# Potential role for nectin-4 in the pathogenesis of pre-eclampsia: a molecular genetic study

**DOI:** 10.1186/s12881-018-0681-y

**Published:** 2018-09-14

**Authors:** Mayuko Ito, Haruki Nishizawa, Makiko Tsutsumi, Asuka Kato, Yoshiko Sakabe, Yoshiteru Noda, Akiko Ohwaki, Jun Miyazaki, Takema Kato, Kazuya Shiogama, Takao Sekiya, Hiroki Kurahashi, Takuma Fujii

**Affiliations:** 1 Department of Obstetrics and Gynecology, Fujita Health University School of Medicine, 1-98 Dengakugakubo, Kutsukake, Toyoake, Aichi 470-1192 Japan; 2 Division of Molecular Genetics, Institute for Comprehensive Medical Science, Fujita Health University, Toyoake, Japan; 3 Division of Morphology and Cell Function, Faculty of Medical Technology, Fujita Health University School of Health Sciences, Toyoake, Japan

**Keywords:** Nectin-4, Pre-eclampsia, Trophoblast

## Abstract

**Background:**

Nectins are cell adhesion molecules that play a pivotal role in adherens junctions and tight junctions. Our previous study using whole-genome oligonucleotide microarrays revealed that nectin-4 was upregulated in pre-eclamptic placentas. We investigated the role of nectin-4 in the etiology of pre-eclampsia.

**Methods:**

We investigated the expression of nectin-4 using real-time RT–PCR, western blot and immunostaining. Additionally, we performed matrigel invasion assay and cytotoxicity assay using cells overexpressing the nectin-4.

**Results:**

*NECTIN4* transcripts were elevated in pre-eclamptic placentas relative to uncomplicated pregnancies. Nectin-4 protein levels in pre-eclamptic placentas were higher on a semi-quantitative western blot. Nectin-4 was localized at the apical cell membrane in syncytiotrophoblast cells and not at the adherens junctions. Nectin-4 was also detected in cytotrophoblasts and a subset of cells in the decidua. Nectin-4 overexpressing trophoblast cells migrated normally in the matrix. However, Natural killer (NK) cells showed a strong cytotoxic effect against nectin-4 overexpressing trophoblast cells. No causative genetic variation was evident in the *NECTIN4* gene from a pre-eclamptic placenta.

**Conclusions:**

There are as yet unknown factors that induce nectin-4 overexpression in trophoblast cells that may contribute to abnormal placentation via an aberrant immune response and the onset of a pre-eclamptic pregnancy.

**Electronic supplementary material:**

The online version of this article (10.1186/s12881-018-0681-y) contains supplementary material, which is available to authorized users.

## Background

Pre-eclampsia is one of the most common obstetrical problems and accounts for almost 15% of pregnancy-associated disorders. Pre-eclampsia is simply defined by a pregnancy-induced hypertension with proteinuria [1], but is actually not a simple disorder as it involves multiple organs including the liver, kidneys, lungs, coagulation and neural systems. There is now an emerging consensus that pre-eclampsia is a complex polygenetic trait in which maternal and fetal genes, as well as environmental factors, contribute to its onset [2–4]. Accumulating evidence has also now indicated that the aberrant expression of placental microRNAs is involved in the pathogenesis of pre-eclampsia [5, 6]. Further to this, the pathogenetic processes underlying this disorder involve numerous factors such as oxidative stress, endothelial dysfunction, vasoconstriction, metabolic changes, thrombotic disorders and inflammatory responses, although the precise mechanisms have remained elusive [7, 8].

Nectins are Ca2 + −independent, immunoglobulin- like cell adhesion molecules that play a pivotal role in both adherens junctions and tight junctions [9]. Nectins were originally described as homologs of poliovirus receptor-like receptors (PVRL) [10]. Some of these molecules interact with the domain of the filamentous-actin (F-actin)-associated molecule (known as afadin) and are involved in signal transduction [11]. Although nectin-1, 2 and 3 have been biologically well characterized as adhesion molecules, the functional properties of nectin-4 are still unknown. Recently, nectin-4 has been demonstrated to be an epithelial receptor for the measles virus [12]. In addition, since certain cancers, (breast, lung and ovarian cancer) overexpress nectin-4, it has been described as a new biomarker for these cancer types [13–15].

We previously performed gene expression profiling of placental tissue from women with and without pre-eclampsia using whole-genome oligonucleotide microarrays [16, 17]. Our results from these analyses subsequently identified ~ 150 genes with significantly altered expression in pre-eclampsia. *NECTIN4* was identified as one of the most significantly upregulated of these genes in pre-eclamptic placentas. In our current study, we have examined the localization of the nectin-4 protein in placental tissues. We then compared the nectin-4 protein levels between women with uncomplicated pregnancies and women with severe pre-eclampsia. Further, we performed some functional assays using nectin-4 overexpressing trophoblast cells. Our present findings provide some additional insights into the role of nectin-4 in the etiology of pre-eclampsia.

## Methods

### Samples

All of the clinical samples analyzed in this study were collected at the Department of Obstetrics and Gynecology, Fujita Health University Hospital, Japan. Placental biopsy samples were obtained during Caesarean sections from both normotensive pregnancy (*n* = 20) and those with severe pre-eclampsia (*n* = 20). Severe pre-eclampsia was defined as a systolic blood pressure of 160 mmHg or higher, or diastolic blood pressure of 110 mmHg or higher on two occasions at least 4 h apart while the patient is on bed rest (unless antihypertensive therapy is initiated before this time) [18]. Normotensive subjects were matched for maternal age, gestational age, and maternal body mass index at pre-pregnancy. In addition to the normotensive term subjects, we collected preterm normotensive control samples from pregnancies with a premature rupture of the membrane due to a breech presentation or a previous caesarean section without evidence of intrauterine infection. The early gestation placental tissues were obtained from elective surgical terminations of pregnancies. Clinical details of these subjects are presented in Table 1.

**Table 1:** Characteristics of the subjects with normotensive pregnancies and severe pre-eclampsia

	Normotensive Pregnancy	Severe pre-eclampsia	*P* Value
	*N* = 25	*N* = 22	
Maternal age (y)	29.4 ± 5.8^a^	30.2 ± 3.6	n.s.
Gestational age (wks)	33.2 ± 5.1	32.9 ± 3.8	n.s.
Parity (n)	0.71 ± 0.76	0.22 ± 0.42	*P* < 0.05
Systolic BP (mmHg)	113.0 ± 10.0	169.6 ± 14.5	*P* < 0.05
Diastolic BP (mmHg)	66.8 ± 9.8	105.9 ± 13.9	*P* < 0.05
Proteinuria^b^	0 (0%)	22 (100%)	*P* < 0.05
Body mass index (BMI)^c^	22.3 ± 4.6	22.3 ± 7.5	n.s.
Placental weight (g)	480.0 ± 244.1	312.9 ± 126.7	*P* < 0.05
Birth weight (g)	1984.9 ± 905.7	1585.5 ± 722.6	*P* < 0.05
Birth weight coefficient	1.012 ± 0.129	0.742 ± 0.215	*P* < 0.05
Hb (g/dl)	10.9 ± 0.9	12.0 ± 1.9	n.s.
Platelet count (× 10^4^/ul)	25.2 ± 3.8	15.4 ± 8.2	*p* < 0.05
GOT (IU/l)	18.3 ± 7.5	39.5 ± 31.7	*p* < 0.05
GPT (IU/l)	15.4 ± 16.4	25.7 ± 15.8	n.s.
LDH (IU/l)	206.8 ± 36.1	333.4 ± 98.1	*p* < 0.05

*Hb* Hemoglobin, *GOT* Glutamate oxaloacetate transaminase, *GPT* Glutamate pyruvate transaminase, *LDH* Lactate dehydrogenase

^a^Data are given as the mean ± standard deviation (SD)

^b^≥2 g in a 24 h collection

^c^pre-pregnancy

To avoid any possible effects of labor on *NECTIN4* expression, only placental samples that were obtained through Caesarean section from women who had not undergone labor were analyzed. A central area of chorionic tissue was dissected and the maternal deciduas and amnionic membranes were removed from these samples. After vigorous washing of the maternal blood with saline, tissues were immediately frozen in liquid nitrogen and stored until use. Informed consent was obtained from each patient and this study was approved by the Ethical Review Board for Clinical Studies at Fujita Health University.

### Real-time RT–PCR

Total RNA was extracted from chorionic villous tissue samples using an RNeasy mini-kit (Qiagen, Valencia, CA), according to the manufacturer’s instructions. To quantify the *NECTIN4* gene expression levels, we performed real-time RT-PCR analyses using an ABI PRISM 7700 Sequence Detection System (Perkin-Elmer, Foster City, CA). A Superscript First-strand Synthesis System for RT-PCR (Invitrogen, Grand Island, NY) using random primers was employed to produce single strand cDNA from total RNA. PCR primers and TaqMan probes (Hs00363974_m1) were obtained from Applied Biosystems GmbH (Weiterstadt, Germany). The housekeeping gene 18S rRNA (Hs99999901_s1) was used to normalize mRNA concentrations, since expression of other genes widely used as controls are often regulated by estrogen. RT-PCR reactions were performed in triplicate using a TaqMan EZ RT-PCR Kit (Perkin-Elmer) in a final volume of 25 μl. The cycling conditions were 2 min at 50 °C, 30 min at 60 °C, and 1 min at 95 °C for RT, followed by 40 cycles of 15 s at 95 °C, and 1 min at 60 °C for PCR amplification.

### Antibodies

Polyclonal goat antibodies raised against the C-terminal cytoplasmic domain of human nectin-4 was used for immunostaining and western blotting (AF2659; R&D, Minneapolis, MN). In addition, rabbit polyclonal anti-human nectin-4 antibodies were raised against amino acid residues 399–415 (CRRLHSHHTDPRSQPEES) and residues 463–479 (CPGSGRAEEEEDQDEGIK) within the cytoplasmic domain of human nectin-4. The resulting antisera were affinity purified on columns coupled to the peptide. To detect cells of the trophoblast lineage, anti-pancytokeratin monoclonal antibodies (Nichirei, Tokyo, Japan) were used.

### Western blot analysis

Total cell lysates from placental tissue were prepared using T-PER (Pierce, Rockford, IL) and the proteins were separated by SDS-PAGE in 14% Tris-glycine gels (TEFCO, Tokyo, Japan). After electrophoresis, the proteins were blotted onto a nitrocellulose membrane and blocked with 5% skimmed milk powder diluted in Tris-buffered saline (TBS) with 0.05% Tween20. Membranes were incubated with diluted antibody preparations overnight at 4 °C. After washing the next day, membranes were incubated with horseradish peroxidase (HRP)-conjugated affinity-purified donkey anti-rabbit or anti-goat IgG antibody (Jackson ImmunoResearch Laboratories, West Grove, PA) for 1 h at room temperature. The blots were then developed using SuperSignal (Pierce) and images were captured using Light-Capture with a cooled CCD camera (ATTO, Tokyo, Japan). MagicMark XP Western Protein Standard (Invitrogen) and Precision Plus Protein Standards (BIO-RAD) were used as size markers. Recombinant human nectin-4 (H00081607-Q01, Abnova) and nectin-4293 T cell transient overexpression lysate (H00081607-T01, Abnova) were also used. For semi-quantitative analysis, the membranes were reprobed with an anti-β-actin antibody (Sigma-Aldrich, St. Louis, MO) as an internal control for the relative loading of the samples.

### Immunostaining

Placental samples were fixed with 10% formaldehyde overnight, and then embedded with paraffin. Tissue sections (2 μm) were placed on silane-coated slides and dried in a conventional oven at 60 °C for 24 h. After deparaffinization and rehydration, endogenous peroxidase activity was blocked with 0.3% hydrogen peroxide in methanol. Polyclonal goat or rabbit antibodies against human nectin-4 were used for detection. Bound antibodies were detected with a peroxidase-based method using Simple Stain MAX-PO (Nichirei, Tokyo, Japan) with 3,3-diaminobenzidine as a substrate. Non-specific goat or rabbit IgG (Zymed, San Francisco, CA) was used as the negative control. Counterstaining was performed with Mayer’s hematoxylin solution. We performed immunostaining for 8 placental samples from pre-eclampsia and 8 controls. We stained three sections for each sample, and we examined 5 fields per each section. The immunostained specimens were examined by an observer who was not aware of the expression data and clinical outcomes. The signal intensities were evaluated by a method proposed previously [19].

### Overexpression of NECTIN4

The cDNA encoding human *NECTIN4* was chemically synthesized and cloned into the pCMV-3Tag-8 vector. HTR-8/SVneo, a widely-used first trimester human trophoblast cell line, was purchased from Dr. Charles Graham at Queen’s University (Kingston, Ontario, Canada). The vector was transfected into HTR-8 cells using Lipofectamine 3000 (Thermo Fisher Scientific). Clones stably overexpressing the *NECTIN4* gene were selected using 200 μg/ml hygromycin. Expression levels were assayed by western blotting with anti-FLAG or anti-nectin-4 antibodies.

### Matrigel invasion assay

HTR-8/SVneo cells overexpressing the *NECTIN4* gene or negative control were seeded into the upper chamber of a 24-well transwell plate with an 8.0-μm pore size coated with Matrigel Basement Membrane Matrix (BioCoat Matrigel Invasion Chamber, Corning). Cells remaining in the upper chamber were removed using a cotton swab and the membranes were inverted and stained with toluidine blue and quantified. The invasion index was calculated, in accordance with the manufacturer’s instructions, as the ratio of the cells invading through the matrigel membrane to those migrating through the control membrane.

### Cytotoxicity assay by NK cell line

NK-92MI were purchased from the ATCC (Manassas, VA). NK-92MI, an IL-2-independent natural killer cell line, is cytotoxic to a wide range of cells. To evaluate the level of cytotoxity, we used a CytoTox 96® Non-Radioactive Cytotoxicity Assay (Promega, Madison, WI). In brief, HTR-8/SVneo cells overexpressing the *NECTIN4* gene or a negative control were prepared as target cells at a density of 2.0 × 10^5^ cells/ml. NK-92MI cells were prepared at a density of 5.0 × 10^5^ cells/ml as effector cells and added to the target cell culture cells at a 2.5:1 effector-to-target (E:T) ratio. The cells were then co-cultured for 4 h at 37 °C. Cell lysis was detected by the release of lactate dehydrogenase (LDH) using an LDH cytotoxicity assay kit. The percentage of cytotoxicity was calculated in accordance with the manufacturer’s instructions (Promega).

### Genomic sequencing of NECTIN4 gene

Genomic DNA was extracted from the placentas using a commercially available kit following the manufacturer’s protocol (Qiagen, Frankfurt, Germany)*.* A total of 41 pre-eclampsia samples and 28 control samples from a normal pregnancy were used. The *NECTIN4* gene ([hg19] chr1:161,040,781-161,059,385) is about 18 kb in size and consists of 9 exons. We divided the entire *NECTIN4* genomic region into three subregions that were amplified using specific primers and KAPA Long Range HotStart DNA Polymerase (KAPA Biosystems, Wilmington, MA). The primers used in this study are listed in Table 2. Long-range PCR was performed under the following conditions: initial denaturation of 95 °C for 3 min followed by 35 cycles of 94 °C for 15 s, 62 °C for 15 s and 68 °C for 8 min. The three PCR products were mixed to equimolar concentrations. Pooled DNA libraries were then prepared using a Nextera XT DNA Sample Preparation Kit according to the manufacturer’s protocol (Illumina, San Diego, CA) and sequenced using MiSeq Reagent Kit v2 (300 cycles) with 150 bp paired-end sequencing (Illumina). Sequence reads were mapped to the human reference sequence (RefSeq: NM_030916.2). The identified variants were confirmed by Sanger sequencing.

**Table 2:** PCR primers used for genomic sequencing

Positions	Forward primers	Reverse primers
chr1:161,040,096-161,047,207	GACTAGAGGACCTTTAAATCCTCCC	GGGTTCTCAGCAGATTGGTGAGATG
chr1:161,046,599-161,054,779	GCCTGATTTTTCAAAGGTGTAACAATCCTAC	CCTTTCTCACCCCAGACTCACCCACATA
chr1:161,054,085-161,059,885	ATGATTCCGTGTGCATGTCACACCCT	GTCTGAGCAAACCATGAGACTAAGT

### Statistical analysis

Intergroup comparisons were made using the Student’s *t* test or one way analysis of variance method and *P* values of less than 0.05 were considered statistically significant.

## Results

### NECTIN4 mRNA and protein are upregulated in a pre-eclamptic placenta

**Fig. 1:**
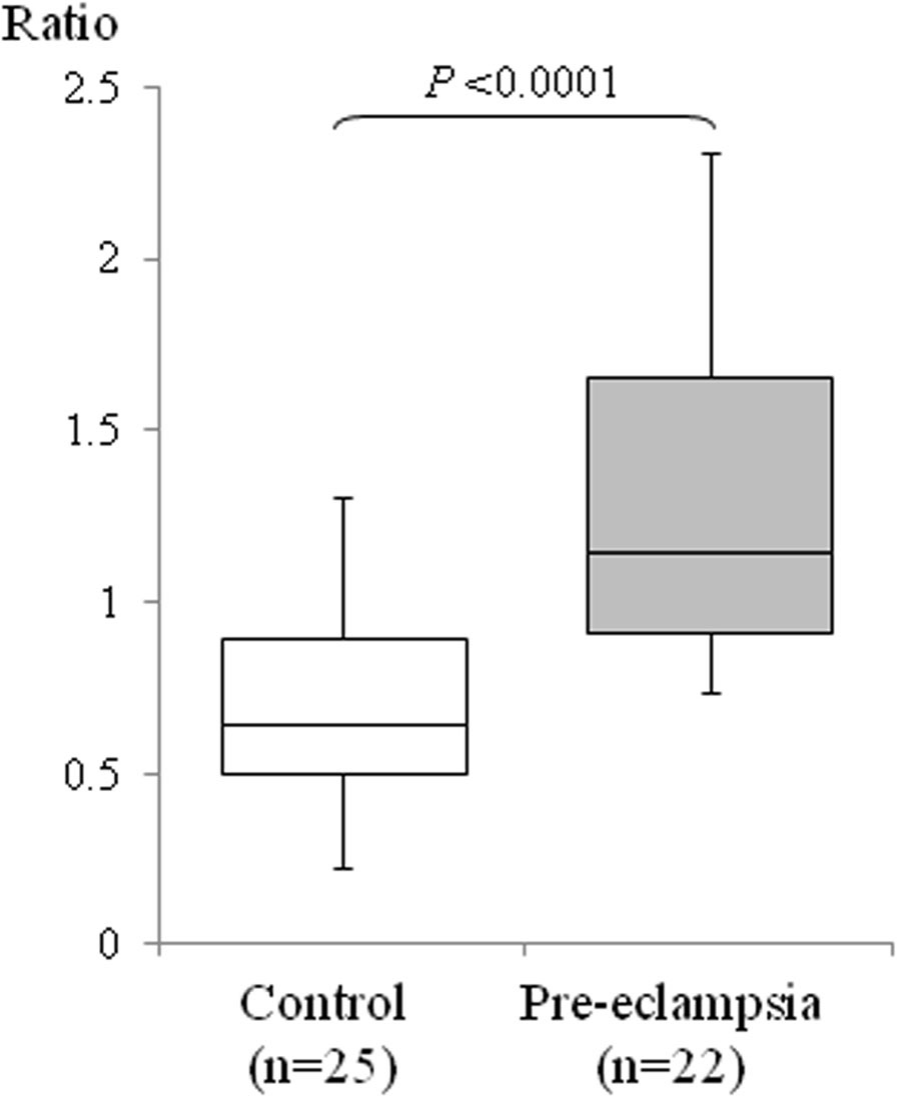
Quantitative PCR analysis of *NECTIN4*. Data were compared for uncomplicated normotensive pregnancy (open bars) versus severe pre-eclampsia (grey bars). The boxes indicate the 25th and 75th percentiles, whilst the bands near the middle indicate the median values. The bars indicate 1.5 interquartile ranges with the outliers specifically marked (x)

**Fig. 2:**
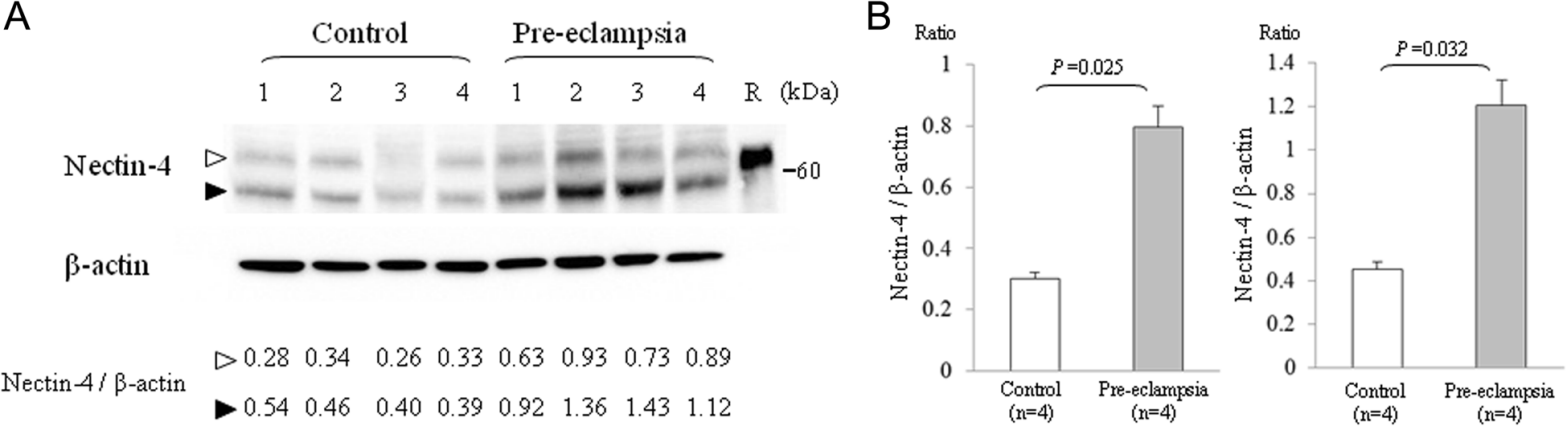
Semi-quantitative western blot analysis of nectin-4. **a** The same blot was incubated against a nectin-4 antibody (upper panel) and a β-actin antibody (lower panel). Rabbit polyclonal anti-human nectin-4 antibodies we raised in this study were used. Lanes 1–4, lysates of placental tissue from uncomplicated normotensive pregnancies; lanes 5–7, lysates of placental tissue from pre-eclamptic pregnancies; lane R, recombinant nectin-4. The data of western blot using commercial goat polyclonal antibodies was shown in Additional file 1: Figure S1. **b** Semi-quantitative analysis of the blot described in A. Data obtained from uncomplicated normotensive pregnancy (open bars) versus severe pre-eclampsia (grey bars) were compared. The left and right panels indicate the data for the upper bands (open triangle), and lower bands (close triangle), respectively. Each bar represents the mean value. Error bars indicate the standard deviation

We performed quantitative RT-PCR for *NECTIN4* using 20 placental samples from severe pre-eclampsia patients and 20 from subjects with an uncomplicated pregnancy. The *NECTIN4* transcript level was found to be significantly increased in placentas from the pre-eclampsia cases (Fig. 1, Additional file 1: Figure S1). We performed western blotting to then examine the expression of nectin-4 protein. Although the molecular weight of nectin-4 is 55.5 kDa based upon its amino acid sequence, total cell lysates from placental samples showed two expressed nectin-4 isoforms of 55.5 kDa and ~ 75 kDa (Fig. 2a, left panel). To confirm that these two species were derived from nectin-4, we used a different nectin-4 antibody and obtained similar immunoblotting results (Additional file 2: Figure S2). To evaluate the nectin-4 protein level, the western blots were subjected to semi-quantitative analyses using β-actin as an internal control. Both isoforms of the nectin-4 protein were found to be more abundant in pre-eclamptic placentas (Fig. 2b).

### Nection-4 protein is produced by syncytiotrophoblasts in the placenta

**Fig. 3:**
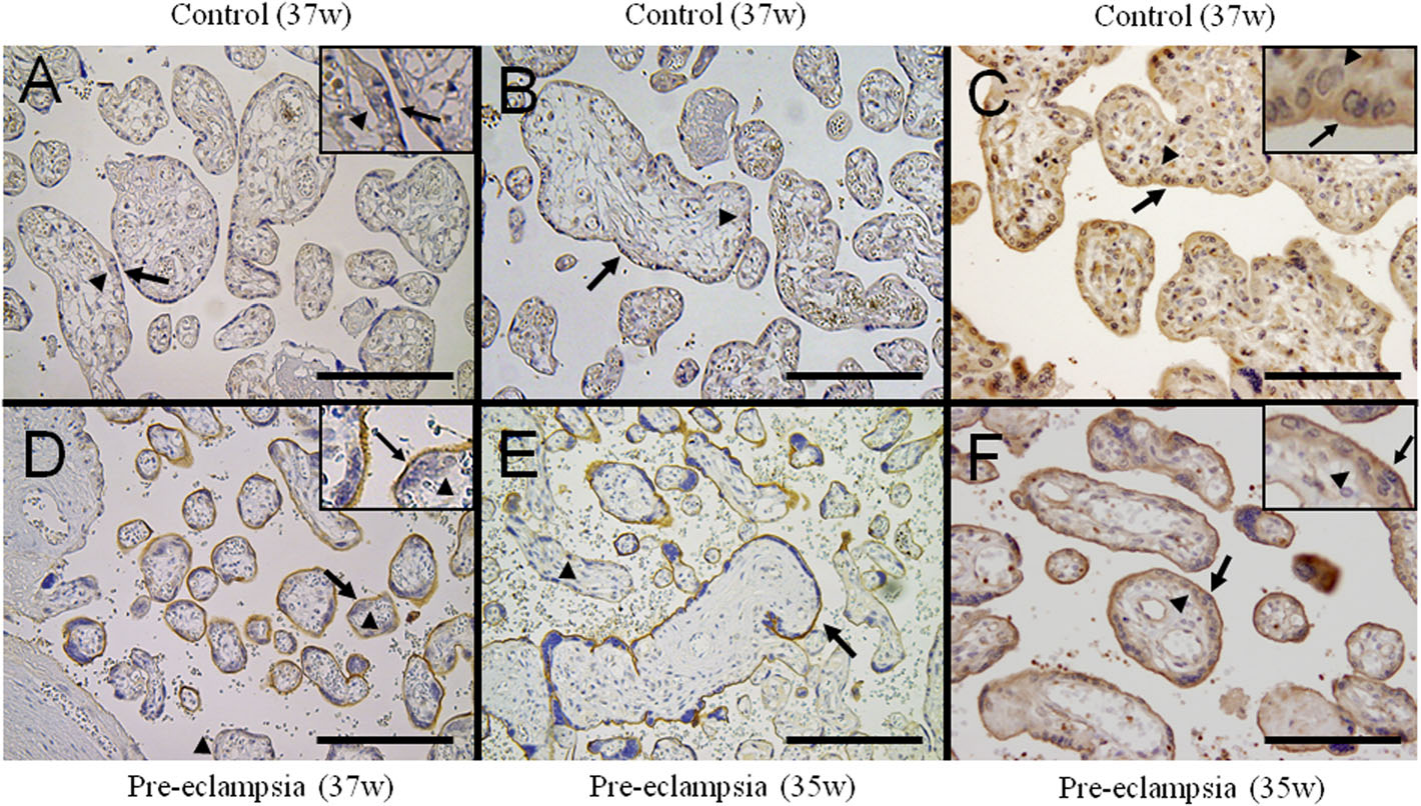
Nectin-4 immunostaining of placental tissue sections. Polyclonal rabbit antibodies raised against the cytoplasmic domain of the human nectin-4 (**a**, **b**, **d** and **e**) or polyclonal goat antibodies raised against the C-terminal domain of the human nectin-4 (**c** and **f**) were used. Positive staining was apparent in the cytoplasm and cell membrane at the apical side of the syncytiotrophoblasts (arrows). Cytotrophoblasts were also positive (arrowheads, insets). The nectin-4 signals were much stronger in pre-eclampsia (**d**, **e** and **f**) compared with uncomplicated normotensive pregnancy (**a**, **b**, and **c**). Original magnifications **a-f**: 400 x. Scale bars, 100 μm

To localize the nectin-4 protein within the placental tissue, we examined placental histological specimens stained with a rabbit polyclonal anti-nectin-4 antibody of confirmed specificity on a western blot. This analysis revealed signals in the cytoplasm and stronger signals at the cell membrane on the apical side in the syncytiotrophoblast cells within the chorionic villi from uncomplicated term pregnancies (Fig. 3a and b, Additional file 3: Figure S3). Similar observations were made using alternate goat anti-nectin-4 antibodies (Fig. 3c). In pre-eclamptic placentas however, the nectin-4 protein signals in the syncytiotrophoblasts were stronger than those detected in uncomplicated pregnancies. Notably, the signal intensities at the apical cell membranes of the syncytiotrophoblast cells in the pre-eclamptic subjects were much stronger than those in the normal pregnancy controls (Fig. 3d, e and f).

**Fig. 4:**
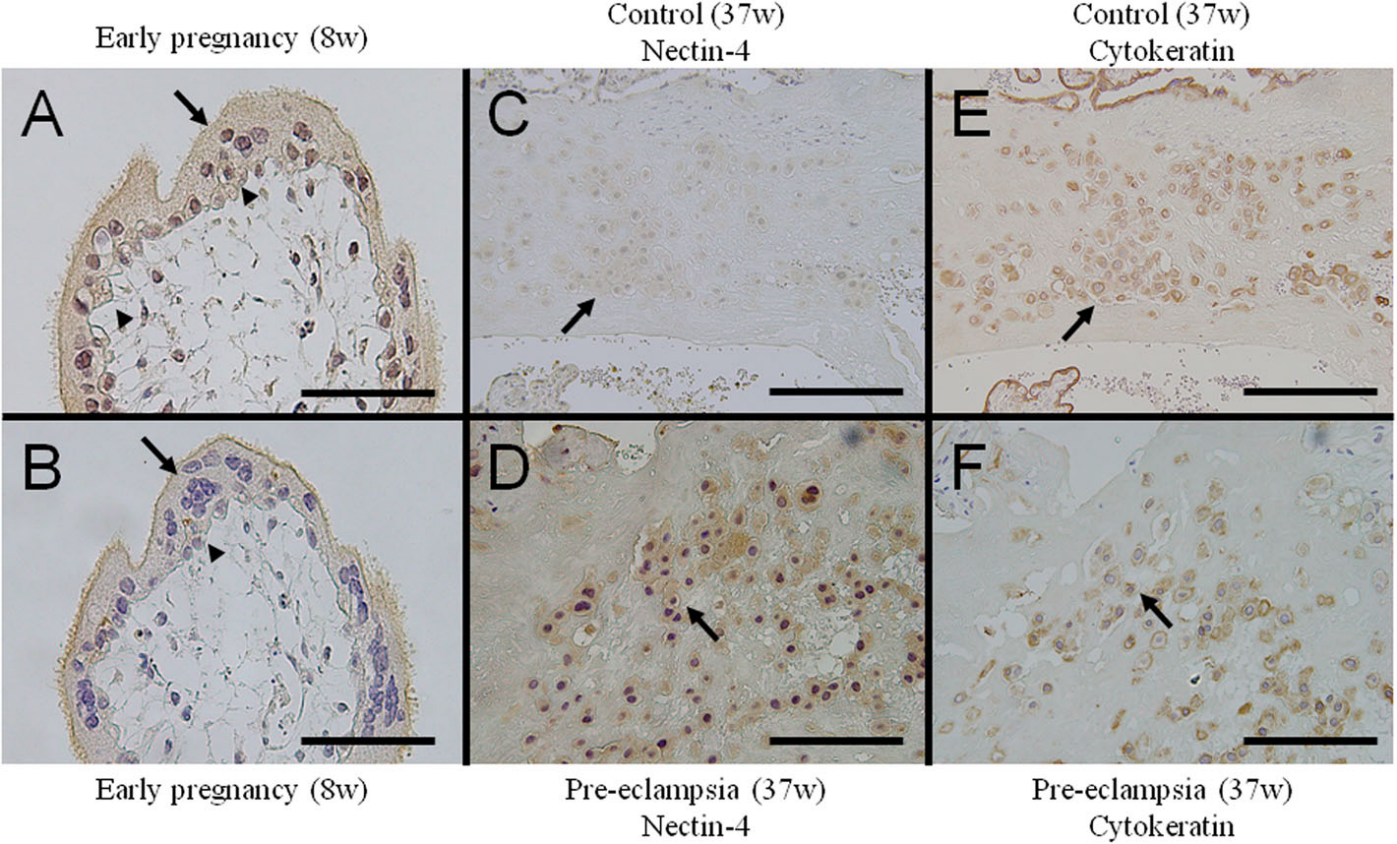
Nectin-4 immunostaining of tissue sections. Chorionic villi from early gestation was stained with polyclonal goat (**a**) or rabbit (**b**) antibodies against human nectin-4. Signals are observed in the syncytiotrophoblast (arrow) and cytotrophoblast cells (arrowhead). Maternal decidua was also examined using goat polyclonal antibodies against human nectin-4 (**c** and **d**). A subset of cells, possibly extravillous trophoblasts, is positive for nectin-4 staining (arrows). To detect cells of the trophoblast lineage, anti-pancytokeratin monoclonal antibodies were used (**e** and **f**). Original magnifications **a-f**: 400 x. Scale bars, 100 μm

Cytotrophoblast cells were also positive for the nectin-4, which was more prominent in samples from early gestation (Fig. 4a and b). Further, we observed the presence of nectin-4-positive cells in maternal decidua (Fig. 4c and d). These cells might be extravillous trophoblasts since they were also positive for cytokeratin (Fig. 4e and f). The signal intensity was stronger in samples from pre-eclampsia than from controls (Fig. 4c and d).

### Nectin-4 protein overexpression does not affect cell migration

**Fig. 5:**
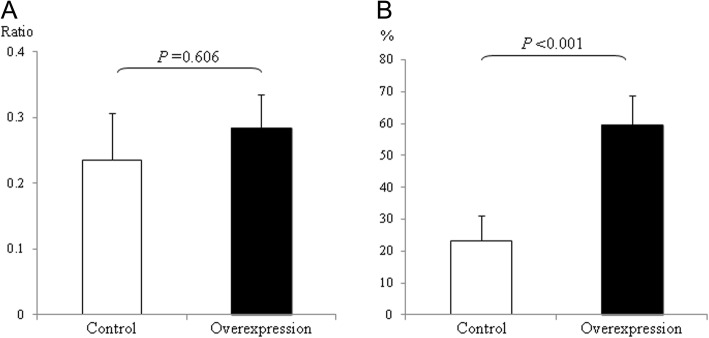
Overexpression of the *NCTIN4* gene in HTR-8 cell lines. **a** Invasion assay. Vertical axis indicates the ratio of cells invading through the matrigel membrane to those migrating through the control membrane. **b** NK cell cytotoxicity assay. Vertical axis indicates the percentage cytotoxicity calculated using an LDH cytotoxicity assay. Data obtained from control HTR-8 cells (open bars) versus HTR-8 cells overexpressing nectin-4 (black bars) were compared. Each bar represents the mean value. Error bars indicate the standard deviation

Since HTR-8/SVneo cells do not strongly express *NECTIN4* (data not shown), we produced a nectin-4 overexpressing HTR-8/SVneo cell line to use as a model system for invasive trophoblasts in pre- eclampsia (Additional file 4: Figure S4). In the early gestational period when placentation occurs, fetal trophoblasts that have invaded the basal membrane of the maternal uterus and then migrate to the uterine interstitium. These cells then migrate to the uteroplacental spiral artery and finally replace the maternal endovascular endothelial cells [20]. It has been proposed that the failure of this vascular remodeling system may lead to the onset of pre-eclampsia [20]. We compared the cell migration ability of trophoblasts overexpressing nectin-4 relative to control trophoblasts. However, the up-regulation of *NECTIN4* did not affect the invasiveness of the HTR-8 cells in the basal membrane-type extracellular matrix (Fig. 5a).

We also speculate that invading fetal trophoblasts may be a target for the maternal immune system, such as NK cells, and that the migration of these cells might be strictly regulated by natural immunity. We thus compared NK cell cytotoxicity towards trophoblasts overexpressing nectin-4 relative to control trophoblasts. Trophoblasts overexpressing nectin-4 showed more sensitivity to the cytotoxic effects of NK cells (Fig. 5b).

### The NECTIN4 gene is not mutated in pre-eclamptic placental tissue

Given the possibility that the up-regulation of *NECTIN4* might be the primary etiology of pre-eclampsia, we hypothesized that mutations in this gene may underlie its up-regulation. We sequenced an 18 kb genomic region that contains the promoter region and 9 exons of the *NECTIN4* gene but found no rare variants in placental samples from pre-eclampsia patients (data not shown).

## Discussion

We have analyzed the overexpression of the *NECTIN4* gene, both at the RNA and protein levels, in pre-eclamptic and normal placentas to test whether alterations in the expression of this gene underlie the etiology of pre-eclampsia. Although other members of the nectin family are expressed ubiquitously, *NECTIN4* is specifically expressed in the skin, esophagus, urinary bladder and placenta. The nectins are known to be adhesion molecules that may have key functions at adherens junctions and tight junctions [9]. However, our current data indicate that the nectin-4 protein is localized at the apical cell membrane of syncytiotrophoblasts, suggesting different functional properties from other nectins. Further, we observed the presence of nectin-4-positive cells in maternal decidua, which might be extravillous trophoblasts. It is thus feasible to suspect a functional linkage between nectin-4 and trophoblast invasion in the placental bed and in maternal uteroplacental arteries [20]. We do not know however if invasive trophoblasts actually express nectin-4 in the plasma membrane during early gestation. We hypothesize from our current analysis that trophoblast invasion is regulated by the surface expression of nectin-4 on these cells.

It has long been accepted that placentation is abnormal in pre-eclampsia [21]. Trophoblast invasion of the interstitial uterine compartment is frequently shallow, and invasion into the spiral artery is often incomplete, during a pre-eclamptic pregnancy. Likewise, the impaired invasion of trophoblasts is believed to underlie the etiology of this disorder. Our current results indicate that nectin-4 expression is upregulated at the apical cell membrane of the syncytiotrophoblasts in pre-eclampsia. The overexpression of nectin-4 in extravillous trophoblast in maternal decidua might reflect invasive trophoblast in early gestation. We hypothesized that trophoblast invasion might be impaired in pre-eclampsia by the overexpression of *NECTIN4*. However, an invasion assay using HTR-8 cell lines did not support this possibility.

In further experiments however, HTR-8 cells stably overexpressing exogenous *NECTIN4* were significantly more sensitive to NK cell cytotoxicity than control HTR-8 cells. These data suggest the possibility that the nectins might regulate NK-target interactions and that the impairment of these interactions might induce the onset of pre-eclampsia. Consistently, there is some evidence that nectins play an important role in the recognition of target cells by NK cells. It has been reported previously that NK cells recognize and kill human glioblastoma cells that express high levels of nectin-2 [22]. Another study has demonstrated that the interaction of nectin-2 and its ligand inhibits NK cell cytotoxicity [23]. Similarly, it has been reported that the interaction of nectin-4 and its ligand might activate NK cell cytotoxity towards trophoblasts, leading to their impaired invasion [24, 25].

It was demonstrated recently that mutations in *NECTIN4* resulted in ectodermal dysplasia-syndactyly syndrome in humans [26, 27]. This is a rare autosomal recessive disorder that might be caused by a loss-of-function of *NECTIN4*. We thus hypothesized that this disorder and pre-eclampsia might represent allelic heterogeneity and the mutation in *NECTIN4* may cause its overexpression of *NECTIN4* and lead to the onset of pre-eclampsia. However, our current data did not reveal any *NECTIN4* variants in pre-eclamptic placentas. Taken together, our present data suggest the possibility that some unknown factors induce nectin-4 protein overexpression in trophoblasts that might cause abnormal placentation via aberrant immunity, and ultimately a pre-eclamptic pregnancy. On the other hand, it is also possible that the elevated expression of the nectin-4 is secondary to the dysfunction of syncytiotrophoblast cells.

## Conclusion

Our current data highlight an old proposition i.e. the direct involvement of NK cells in the etiology of pre-eclampsia. Further detailed analysis of the functional roles of nectin-4 in placentation will be needed to properly assess the association of its overexpression with the etiology of pre-eclampsia.

## Additional files

Additional file 1: **Figure S1.** Quantitative PCR analysis of *NECTIN4*. Data were compared for uncomplicated normotensive pregnancy (open bars) versus severe pre-eclampsia (grey bars). Among the uncomplicated normotensive pregnancy, data were also compared for pre-term (≤36wks) versus term (≥37wks). The boxes indicate the 25th and 75th percentiles, whilst the bands near the middle indicate the median values. The bars indicate 1.5 interquartile ranges with the outliers specifically marked (x). (TIF 2224 kb)

Additional file 2: **Figure S2.** Western blot analysis of nectin-4. Polyclonal goat antibodies raised against the C-terminal cytoplasmic domain of human nectin-4 were used (R&D). The bands indicated by the open and closed triangles correspond with those shown in Fig. 2. (TIF 2218 kb)

Additional file 3: **Figure S3.** Negative control for immunostaining. Non-specific polyclonal IgG was used. (TIF 11898 kb)

Additional file 4: **Figure S4.** Western blot analysis of cell lines with overexpression of nectin-4. Left panel indicates the data for anti-FLAG antibodies, while right panel indicates the data for anti-nectin 4 antibodies. Lane N, negative control (no transfection); lane P, positive control (transient expression); lane O, stable line with overexpression of nectin-4. (TIF 3354 kb)

## Abbreviations

F-actin: Filamentous-actin
HRP: Horseradish peroxidase
LDH: Lactate dehydrogenase
NK: Natural killer
PVRL: Poliovirus receptor-like receptors
RT-PCR: Reverse transcription polymerase chain reaction
TBS: Tris-buffered saline
